# Complete genome sequence of *Trinickia caryophylli* ATCC25418

**DOI:** 10.1128/mra.00929-24

**Published:** 2025-02-12

**Authors:** Juhyun Kim, Ye Eun Kim, Sung-Jun Hong

**Affiliations:** 1 Plant Quarantine Technology Center, Animal and Plant Quarantine Agency, Gimcheon-si, Republic of Korea; University of Strathclyde, Glasgow, United Kingdom

**Keywords:** *Trinickia caryophylli*, carnation bacterial wilt, plant pathogenic bacteria, genome analysis

## Abstract

*Trinickia caryophylli* causes carnation bacterial wilt disease. We report the complete genome sequence of *T. caryophylli* ATCC25418. There are two circular chromosomes with a total length of 6,589,249 bp, and the total GC content is 64.7%. This study describes the complete genome of strain ATCC25418.

## ANNOUNCEMENT

Carnation bacterial wilt (CBW) is one of the most damaging diseases of carnations (*Dianthus caryophyllus* L.), causing significant production losses due to plant withering (1, 2). CBW is caused by *Trinickia caryophylli*, a gram-negative, soil-borne bacterium belonging to the β-proteobacteria subphylum (3). In this study, we present the complete genome sequence of *T. caryophylli* strain ATCC25418, isolated in 1930 from a carnation plant in the USA.

Strain ATCC25418 was obtained from the American Type Culture Collection in 2023. The strain was cultured on Nutrient agar (BD Difco, USA) with pH 7.2 for 24 h at 30°C under aerobic conditions. A pure colony of ATCC25418 was obtained after several times of restreaking. Genomic DNA was extracted using the Wizard Genomic DNA Kit (Promega). The extracted genomic DNA was separated and sequenced using two NGS platforms to leverage their respective strengths. The gDNA was sequenced using the Pacific Biosciences (Pacbio) Sequel II and the Illumina NovaSeq 6000 by Macrogen (South Korea). A library for NovaSeq sequencing was constructed using the TruSeq Nano DNA high-throughput library preparation kit (Illumina). The HiFi libraries were prepared with the SMRTbell Express Template Prep Kit 2.0 (PacBio) for PacBio sequencing. The gDNA was sheared by a Megaruptor 3 (Diagenode, USA), and size selection was performed using the BluePippin size selection system (cutoff value: <8 kb) after library production. Genome assembly was performed using the microbial assembly application SMRTLink 11.1.0.166339, based on the Hierarchical Genome Assembly (HGAP) version 4 with default parameters (4). To ensure high-quality Illumina reads, we performed an initial quality assessment using FastQC version 0.11.7 (5), which allowed us to visually inspect the read quality. For quality control, reads containing ≤90% of bases at Q30 were filtered out to remove low-quality data. Adapter trimming and read filtering were performed using Trimmomatic version 0.38 with the following parameters (ILLUMINACLIP: Adapter:fasta:2:30:10:8, LEADING:15, TRAILING:15, SLIDINGWINDOW:4:15, and MINLEN:36) (6). The *de novo* assembly was conducted using only Pacbio reads with HGAP, followed by sequence correction using Illumina reads for enhanced accuracy with Pilon version 1.21 with a minDepth parameter of 0.01 (7). Gene prediction and annotation were performed using the NCBI Prokaryotic Genome Annotation Pipeline version 6.5 (8) (Table 1).

**TABLE 1:** The results of the genome sequence of *T. caryophylli* ATCC25418

Category	Feature	ATCC25418
HiFi read statistics	HiFi reads	60,736
	HiFi read bases	549,427,978
	HiFi N50 (bp)	9,046
Illumina data set	Raw read bases	2,098,409,854
	Raw total reads	13,896,754
	Filtered read bases	1,540,088,042
	Filtered total reads	10,202,770
	Read length (nt)	150
	Q30 (%)	97.16
Assembly	Contigs	2
Contig 1	Size (bp)	4,294,889 (circular)
Contig 2	Size (bp)	2,294,360 (circular)
Total data	Genome size (bp)	6,589,249
	*N*_50_	4,294,889
	Depth (×)	83.2
	GC content (%)	64.7
	CDS	5,764
	tRNA	57
	rRNA	12

To confirm the circular structure of the assembled genome, a dot plot analysis with Gepard version 2.1 revealed a continuous overlap between the start and end regions of each assembled contig, indicating a circular conformation (9). The total genome size of *T. caryophylli* (6.59 Mb) falls within the range of the *Trinickia* genus, with a slightly higher GC content (64.7%) than its close relatives (10, 11).

## Data Availability

The complete genome sequence of *T. caryophylli* ATCC25418 is deposited in GenBank under accession numbers CP131477 and CP131478 (BioProject accession PRJNA998194). Seqencing reads are available in the Sequence Read Archive (SRA) under accession numbers SRR31162397 and SRR31221928 for Pacbio and Illumina raw reads, respectively.
